# Loneliness Literacy Scale: Development and Evaluation of an Early Indicator for Loneliness Prevention

**DOI:** 10.1007/s11205-013-0322-y

**Published:** 2013-04-03

**Authors:** Rianne Honigh-de Vlaming, Annemien Haveman-Nies, Inge Bos-Oude Groeniger, Eveline J. C. Hooft van Huysduynen, Lisette C. P. G. M. de Groot, Pieter van’t Veer

**Affiliations:** 1 Division of Human Nutrition, Academic Collaborative Centre AGORA, Wageningen University, Wageningen, The Netherlands; 2 Academic Collaborative Centre AGORA, GGD Gelre-IJssel (Community Health Service), Apeldoorn, The Netherlands

**Keywords:** Loneliness, Health literacy, Psychometrics, Public health, Elderly people

## Abstract

To develop and evaluate the Loneliness Literacy Scale for the assessment of short-term outcomes of a loneliness prevention programme among Dutch elderly persons. Scale development was based on evidence from literature and experiences from local stakeholders and representatives of the target group. The scale was pre-tested among 303 elderly persons aged 65 years and over. Principal component analysis and internal consistency analysis were used to affirm the scale structure, reduce the number of items and assess the reliability of the constructs. Linear regression analysis was conducted to evaluate the association between the literacy constructs and loneliness. The four constructs “motivation”, “self-efficacy”, “perceived social support” and “subjective norm” derived from principal component analysis captured 56 % of the original variance. Cronbach’s coefficient α was above 0.7 for each construct. The constructs “self-efficacy” and “perceived social support” were positively and “subjective norm” was negatively associated with loneliness. To our knowledge this is the first study developing a short-term indicator for loneliness prevention. The indicator contributes to the need of evaluating public health interventions more close to the intervention activities.

**Electronic supplementary material:**

The online version of this article (doi:10.1007/s11205-013-0322-y) contains supplementary material, which is available to authorized users.

## Introduction

Elderly people are at increased risk of loneliness due to age-related life changes such as retirement, loss of a partner, friends or relatives, and physical and mental disabilities. These life changes affect on the one hand the social network ties and on the other hand the social support needs of elderly people, two important factors related to loneliness (Dykstra 2009; Van Tilburg and De Jong Gierveld 2007). Therefore, network development is the most commonly used strategy to reduce the prevalence of loneliness in the community (Cattan et al. 2005; Findlay 2003; Fokkema and Van Tilburg 2007; Schoenmakers et al. 2012; Stevens and Martina 2006). However, to alleviate or prevent feelings of loneliness two other strategies have shown to be important, namely lowering standards regarding relationships and reduction of the importance of the loneliness experience (Fokkema and Van Tilburg 2007; Fokkema and Knipscheer 2007; Van Tilburg 1988).

In the eastern part of the Netherlands, the prevalence of loneliness among elderly people aged 65 years and over is around 40 %, as measured with the Dutch De Jong Gierveld Loneliness Scale (De Jong Gierveld and Kamphuis 1985; De Jong Gierveld and Van Tilburg 1999; GGD Gelre-IJssel 2007). This high prevalence, rapid population ageing and the severity of the problems related to loneliness made local policymakers decide to designate loneliness prevention as one of their priority areas. As a result, the loneliness prevention programme *Healthy Ageing* was developed aiming to reduce the prevalence of loneliness among non-institutionalized elderly people in the community, mainly by stimulating network development.

To evaluate the *Healthy Ageing* programme long-term as well as short-term outcomes need to be investigated. Long-term outcomes can indicate overall effectiveness, whereas short-term outcomes can indicate at an early stage whether intervention activities are well received and potentially effective. Besides, measuring short-term outcomes provides insight in how an intervention works and enables health professionals to adapt and improve the intervention activities in an early stage (Craig et al. 2008; Nutbeam 1998; Victora et al. 2004). So far no validated short-term outcome indicators are available for measuring early results of loneliness interventions, while appropriate long-term outcome indicators for loneliness, social support and network size are frequently used (Dickens et al. 2011; Findlay 2003). Therefore, we aimed to develop an outcome indicator called ‘Loneliness Literacy Scale’ (LLS) in order to be able to evaluate the short-term effects of the loneliness prevention programme *Healthy Ageing* on the level of behavioural determinants. This indicator is based on the literacy aspects motivation and ability to gain access to, understand, and use information to promote and maintain good health, as defined in the outcome model for health promotion of (Nutbeam 1998). Nutbeam stated that health literacy measures include for example health-related knowledge, attitudes, motivation, behavioural intentions, personal skills and self-efficacy (Nutbeam 1998, 2000). More recently, he appealed for the development of literacy indices tailored to specific health topics and contexts (Nutbeam 2009). In this article we describe the development and evaluation of a literacy scale related to loneliness.

## Methods

### Scale Development

For the development of the LLS the Intervention Mapping approach was used (Bartholomew et al. 2011b). Determinants of loneliness were systematically identified during the first two steps of this approach: conduct a needs assessment (step 1) and formulate objectives (step 2). Hereby evidence from literature and experiences from local policy makers, health and welfare workers and representatives of the target group were taken into account.

The needs assessment started with the identification of high risk groups for loneliness. Elderly people with a low discretionary income, with physical restrictions, with mild depressive symptoms and widowed elderly appeared to be lonelier according to literature and data from the local health monitor (GGD Gelre-IJssel 2006).

Thereafter, for each risk group causes for loneliness were identified and transformed to 21 behaviour change objectives. For example, it appeared that elderly with a low discretionary income have little money left for membership-fees, which diminishes their opportunities for social engagement. This resulted in the objective: “Elderly with a low discretionary income apply for financial support for social activities by the local government”. The risk group widowers mainly suffer from emotional loneliness and have to learn how to cope with these feelings. An example of a related objective was: “Aged widowers join social support groups for bereavement.”

Then, the 21 behavioural objectives were summarized and reduced to two main objectives, namely elderly people become or stay socially engaged and search for social support. With social support we refer to different kinds of support such as help in learning how to cope with feelings of loneliness, emotional support to enhance self-esteem, transport services for elderly to support mobility and financial support to facilitate engagement. This support can be derived from both informal support systems e.g. friends and family and from formal support systems e.g. general practitioner, elderly advisor and governmental services (Berkman et al. 2000; Hogan et al. 2002).

Afterwards, behavioural determinants for the behaviours “becoming or staying social engaged” and “searching for support” were identified by studying health behaviour theories such as the Social Cognitive Theory, Theory of Planned Behaviour, Theory of Reasoned Action, and Health Belief Model (Bartholomew et al. 2011a; Michie et al. 2008; Bandura 2006; Fishbein et al. 2001; Noar and Zimmerman 2005; Fishbein 2000; Michie et al. 2005). Eight determinants were perceived to be most relevant, namely: awareness, knowledge, self-efficacy beliefs, skills, attitudinal beliefs, normative beliefs, motivation to comply and intention. For simplification, these determinants were summarized into three general constructs of loneliness literacy. To be consistent with health promoters’ practices, “awareness” was combined with “knowledge”; “skills” was combined with “self-efficacy”; and “attitudinal beliefs”, “normative beliefs”, “motivation to comply” and “intention” were combined in the overall concept “motivation”. The construct “knowledge” addressed factual knowledge and awareness about the availability of municipal services for elderly people with physical or mental health problems. The construct “self-efficacy” covered self-perceived social skills and skills to collect information about municipal services. The construct “motivation” comprised attitudinal beliefs (personal attitude and outcome expectations) and normative beliefs (social norms and motivation to comply). As a result, the construct “motivation” included intrinsic motivation as well as motivation driven by external support.

Next, so called change objectives were formulated for each combination of the two behaviours and the three behavioural determinants for the four priority groups. This resulted in a matrix with more than 200 potential change objectives contributing to the prevention of loneliness. These change objectives were summarized to come to a practical and manageable set. For example, knowledge about where to find information about dealing with bereavement, where to find information about organizations involved with depression prevention and where to find information about living on a low income were merged into knowledge about finding information about physical and mental health problems related to ageing. At the end, 43 change objectives remained.

Finally, each change objective was rephrased into a statement and was included as individual item in the draft version of the LLS. The scale was pre-tested for understandability among a group of seven volunteers from the target population, after which a few improvements were made. In the end, the scale contained 14 items for the construct “knowledge”, 11 items for “self-efficacy” and 18 items for “motivation”. Ten items of the construct “knowledge” were assessed using a dichotomous scale (1 = “no”; 2 = “yes”). All other items of the three constructs were assessed using a 5-point Likert scale (1 = “fully agree” or “definitely”; 5 = “fully disagree” or “definitely not”). See the supplementary material for a description of all items.

### Data Collection

To psychometrically evaluate the scale, a study was carried out among non-institutionalized elderly people aged 65 years and over living in the municipality of Epe, a rural community in the Eastern part of the Netherlands, in 2009. To exploit heterogeneity, participants were selected on the basis of their score for loneliness in the baseline study of the *Healthy Ageing* programme in 2008 (n = 903) as measured with the De Jong Gierveld Loneliness Scale (De Jong Gierveld and Kamphuis 1985; De Jong Gierveld and Van Tilburg 1999; De Vlaming et al. 2010). Persons with the lowest and highest loneliness scores at baseline were selected, resulting in a sample of 203 persons indicated as not lonely (score 0–2) and 193 persons indicated as moderately to very severely lonely (score 6–11). Participants received a paper-and-pencil questionnaire at their home address and were asked to return the questionnaire by post.

### Other Measurements

Besides the 43 loneliness literacy items, the background variables gender, age and marital status were assessed. Data on education level were imported from the baseline dataset. Furthermore, self-perceived health was assessed with the question ‘How do you perceive your health in general?’, using a 5-point Likert scale ranging from excellent to poor.

Loneliness was assessed with the De Jong Gierveld Loneliness Scale consisting of 11 questions of which 5 are positively and 6 negatively formulated. Three answer categories were provided (“yes”, “more or less”, “no”). For the positive items “no” and “more or less” answers were an indication for loneliness (1 point), whereas for the negative items “yes” and “more or less” were an indication for loneliness (1 point). A score of 0–2 corresponds to no loneliness, 3–8 moderate loneliness, 9–10 severe loneliness, and 11 very severe loneliness. The loneliness scale of De Jong Gierveld permits one missing value per subject to which a score of 0 is given (De Jong Gierveld and Kamphuis 1985; De Jong Gierveld and Van Tilburg 1999; Van Tilburg and De Jong Gierveld 1999). The internal consistency of the loneliness scale in this dataset was in line with outcomes in other studies (Cronbach’s coefficient α 0.92) (De Jong Gierveld and Van Tilburg 1999).

### Statistical Analysis

To affirm the underlying scale structure and to reduce the number of scale items, principal component analysis (PCA) with oblique (oblimin) rotation was used (Floyd and Widaman 1995; Suhr 2005). To test the appropriateness of the data for PCA, the underlying assumptions were tested. The Kaiser–Meyer–Olkin index (KMO) of sampling adequacy was >0.7, indicating that patterns of correlations are relatively compact and suitable for PCA. According to Barlett’s sphericity test (χ^2^ = 2,116.43, *df* = 231, *P* < 0.001), multicolinearity and singularity were not violated (Lattin et al. 2003; Field 2005). Internal consistency reliability of the constructs, based on the identified components from PCA, was assessed by Cronbach’s coefficient α, taking a value of ≥0.7 as adequate (Bland and Altman 1997).

Four-, five-, and six- component solutions were compared, of which the four-component solution appeared to be most meaningful. To shorten the LLS, item reduction was achieved by excluding two items with component loadings <0.4, ten items with a high number of missing values and comments of participants suggesting misinterpretation of the questions, and another nine items that hardly contributed to the reliability of the constructs.

Concurrent validity of the LLS was tested in three steps. In advance, a mean score had been calculated for each of the constructs by adding the scores on the filled out items divided by the total number of items per construct, allowing a maximum of one missing value for each construct. For the evaluation, first, literacy scores of not lonely, mildly lonely, severely lonely and very severely lonely participants, based on the data of 2009, were compared using ANOVA. Second, the association between the mean scores of each of the loneliness literacy constructs as independent and loneliness as dependent variable was analysed in separate univariate models (N = 264). Third, the constructs were analysed together in a crude (N = 264) and adjusted (N = 245) multivariate model, including the confounders gender, age, marital status and education. This procedure enabled us to adjust for potential residual correlation between the discovered constructs, which is characteristic of an oblique rotation procedure (Floyd and Widaman 1995). All statistical calculations were performed using SPSS for Windows version 17.0.2.

## Results

### Sample Characteristics

Of 396 invited persons, 303 persons (76 %) completed the questionnaire, 165 persons (81 %) from the not lonely sub-sample and 133 persons (69 %) from the lonely sub-sample. The sample included slightly more women (55 %) than men and 17 % of the participants followed only primary education. Mean age of the study sample was logically one year older at the time of the current study in comparison to baseline, namely 75.5 years. The mean ± SD loneliness score was significantly lower in 2009 compared to 2008 (3.0 ± 3.5 versus 3.6 ± 4.2). At the time of the current study, 58 % of the people were indicated as not lonely, 29 % as moderately lonely, 9 % as severely lonely and 4 % as very severely lonely (Table 1).

**Table 1:** Background characteristics of elderly Dutch study participants (N = 303) at baseline (2008) and after 1 year (2009)

	Baseline study 2008	Current study 2009
Gender (%)		
Men	45.0	As in 2008
Women	55.0	
Education (%)		
No/primary education	17	As in 2008
Low education	48	
Intermediate education	14	
High education	21	
Age		
Mean age (SD)	74.5 (6.7)	75.5 (6.7)
Marital status (%)		
Married or living together	69	68
Widow, widower	24	25
Other living alone	7	7
Loneliness (%)		
Not lonely (0–2)	55	58
Moderately (3–8)	27	29
Severely (9–10)	14	9
Very severely (11)	4	4
Mean score loneliness (SD)	3.6 (4.2)	3.0 (3.5)^a^

^a^Mean difference in loneliness is significant (*P* < 0.01), paired sample *t* test (N = 286)

### Scale Structure and Reliability

The pattern matrix of the four-component solution appeared to be most meaningful and interpretable, and accounted for 56 % of the total variance (Table 2). Items relating to the target behaviours “becoming or staying social engaged” and “searching for support”, initially grouped in the constructs “knowledge”, “self-efficacy” and “motivation” were redistributed by PCA. The theoretical construct “knowledge” is omitted because of the high number of missing values. Items of the theoretical construct “self-efficacy” clustered in one component and accordingly concerned the self-perceived ability to participate in social activities or conversations, to manage gathering information or to ask for support. Items relating to the broad construct “motivation” in our theoretical model were divided over three constructs, namely: “motivation”, “perceived social support” and “subjective norm”. The new construct “motivation” included mainly items about the motivation to search for support. The component “perceived social support” included items about previously experienced social support and the motivation to comply with the opinion of others. The last construct “subjective norm” included items about respondents’ personal opinion and the perceived opinion of family, friends and neighbours with regard to participation.

**Table 2:** Pattern matrix and Cronbach’s coefficient α for loneliness literacy constructs

	Item	Factor loading	Cronbach’s α
Motivation	In my municipality there are professionals who can help people who feel gloomy or lonely	0.826	0.866
	Meetings for bereavement are offered in my municipality	0.817	
	If I felt lonely, I would search for professional help to reduce these feelings	0.805	
	A support group would help me to give ageing problems a place	0.790	
	If I have problems, a conversation with the elderly advisor helps me to solve my problems	0.684	
	If I lost my partner, I would follow a bereavement course	0.646	
Self-efficacy	I can manage in daily living as regards finding information	0.789	0.826
	I feel self-efficacious enough to go to an activity on my own	0.766	
	If I need help from others, I am able to arrange it myself	0.738	
	I am able do almost anything if I really want to	0.709	
	I can manage in daily living as regards arranging transportation to activities	0.658	
	In a group of friends/acquaintances, I speak up regularly	0.646	
Perceived social support	My family is there for me if I ask for help	0.787	0.735
	I perceive my family’s opinion as important	0.742	
	My neighbours are there for me if I ask for help	0.608	
	My friends are there for me if I ask for help	0.585	
	I perceive my neighbours’ opinion as important	0.482	
	I perceive my friends’ opinion as important	0.443	
Subjective norm	My friends think it is important for me to participate in activities	−0.816	0.807
	My family thinks it is important for me to participate in activities	−0.783	
	By participating in activities I remain among men	−0.692	
	My neighbours think it is important for me to participate in activities	−0.532	

The Cronbach’s coefficient α was above 0.7 for each of the four components, thus confirming an adequate internal consistency between the items within a construct: 0.87 for “motivation”, 0.83 for “self-efficacy”, 0.74 for “perceived social support” and 0.81 for “subjective norm” (Table 2).

### Concurrent Validity

Concurrent validity was evaluated by calculating the mean scores for the literacy constructs per loneliness category i.e. not lonely, mildly lonely, severely lonely or very severely lonely (Table 3). The mean scores for the constructs “self-efficacy” and “perceived social support” were higher for people who were lonely than for people who were not lonely. The mean scores for the constructs “motivation” and “subjective norm” did not differ between the loneliness categories. Crude univariate regression analysis confirmed this, demonstrating that the construct “self-efficacy” [β = 2.08 (95 % CI 1.60; 2.58)] and the construct “perceived social support” [β = 1.54 (95 % CI 0.93; 2.14)] were significantly associated with loneliness and explained 21 and 9 % of the variance in loneliness respectively (Table 4, models 0). Thereafter, multivariate analysis was conducted taking the four constructs together in the model (model 1). The constructs “self-efficacy” and “perceived social support” were significantly positively associated with loneliness, meaning that poor literacy scores were related to more severe loneliness. The construct “subjective norm” was significantly negatively associated with loneliness and the construct “motivation” was not associated with loneliness. After adjustment for confounders [model 2) the associations between loneliness and the constructs “self-efficacy” [β = 1.62 (95 % CI 1.11; 2.14)], “perceived social support” [β = 1.27 (95 % CI 0.69; 1.85)] and “subjective norm” (β = −0.59 (95 % CI −0.99; −0.19)) remained significant at *P* < 0.05. “Motivation” was excluded in the final model (model 3) as this construct did not contribute to the explanation of loneliness. In total, 41 % of the variance in the final model was explained; 29 % by the three remaining loneliness literacy constructs and 12 % by the confounders.

**Table 3:** Means (SD)^a^ for loneliness literacy constructs “motivation”, “self-efficacy”, “perceived social support” and “subjective norm” for four categories of loneliness among elderly Dutch participants (2009) (N = 256)

	Motivation	Self-efficacy	Perceived social support	Subjective norm
Loneliness (2009)				
Not lonely (0–2)	2.8 (0.9)	1.6 (0.6)	1.8 (0.6)	2.4 (1.1)
Moderately lonely (3–8)	2.8 (0.9)	2.0 (0.8)	2.0 (0.6)	2.3 (0.9)
Severely lonely (9–10)	3.0 (0.9)	2.4 (0.9)	2.5 (0.6)	2.4 (0.7)
Very severely lonely (11)	2.8 (0.8)	2.9 (1.0)	2.2 (0.8)	2.5 (0.8)
All	2.8 (0.9)	1.8 (0.8)	2.0 (0.7)	2.4 (1.0)

^a^Loneliness literacy scores range from 1 (good/favourable) to 5 (bad/unfavourable)

**Table 4:** Crude univariate and crude and adjusted multivariate regression analysis for the association between loneliness and the health literacy constructs elderly Dutch participants (2009)

Model	Motivation β (95 % CI)	Self-efficacy β (95 % CI)	Perceived social support β (95 % CI)	Subjective norm β (95 % CI)	R^2a^
0	0.11 (−0.37; 0.58)	2.08* (1.60; 2.58)	1.54* (0.93; 2.14)	−0.05 (−0.48; 0.39)	
1	−0.15 (−0.60; 0.31)	1.90* (1.40; 2.39)	1.51* (0.89; 2.12)	−0.59* (−1.03; −0.15)	0.28
2	−0.19 (−0.62; 0.23)	1.62* (1.11; 2.14)	1.27* (0.69; 1.85)	−0.59* (−0.99; −0.19)	0.42
3		1.61* (1.10; 2.13)	1.25* (0.68; 1.83)	−0.66* (−1.03; −0.29)	0.42

Model 0: univariate model with mean scores for “motivation”, “self-efficacy”, “perceived social support” or “subjective norm” as independent variables (N = 256)

Model 1: crude multivariate model including mean score for “motivation”, “self-efficacy”, “perceived social support” and ‘subjective norm” as independent variables (N = 256)

Model 2: adjusted multivariate model including mean scores for “motivation”, “self-efficacy”, “perceived social support”, “subjective norm”, gender, age, marital status and education as independent variables (N = 239)

Model 3: adjusted multivariate model including mean scores for “self-efficacy”, “perceived social support”, “subjective norm”, gender, age, marital status and education as explanatory variables (N = 239)

* Significant at *P* < 0.05

^a^R^2^ = variance explained by the model

**Fig. 1:**
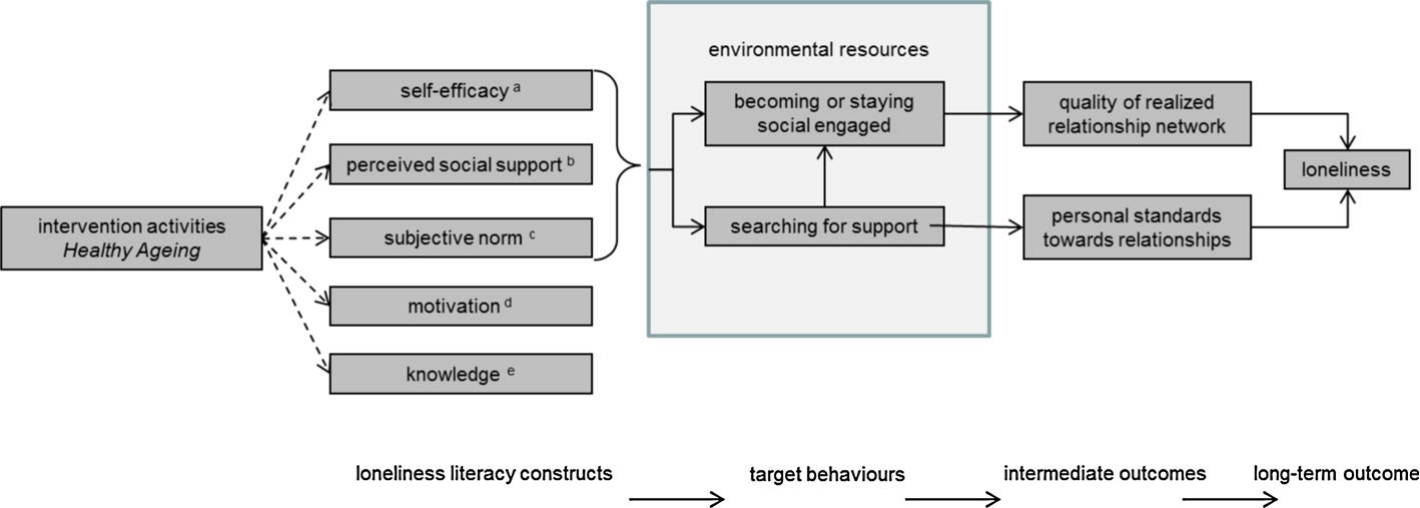
Intervention logic model of the *Healthy Ageing* programme focussing on loneliness literacy. ^a^ Self-efficacy: self-perceived ability to participate in social activities or conversations, to collect information or to ask for support. ^b^ Perceived social support: previously experienced social support and the motivation to comply with the opinion of others. ^c^ Subjective norm: respondents’ personal opinion and the perceived opinion of family, friends and neighbours with regard to participation. ^d^ Motivation: motivation to search for support—not included in final Loneliness Literacy Scale. ^e^ Knowledge: factual knowledge and awareness about the availability of municipal services for elderly people with physical or mental health problems—not included in final Loneliness Literacy Scale

As a result, Fig. 1 represents the intervention logic model of the *Healthy Ageing* programme as derived by the PCA, reliability and regression analysis. The model visualises the relationship between the intervention activities, loneliness literacy, the behaviours “becoming or staying social engaged” and “searching for support” and loneliness.

## Discussion

We aimed to develop a scale to measure short-term outcomes of the loneliness intervention programme *Healthy Ageing.* PCA resulted in the identification of four meaningful constructs, namely “motivation”, “self-efficacy”, “perceived social support” and “subjective norm”. Each of the four constructs had a good internal consistency reliability, indicated by a Cronbach’s coefficient α > 0.7. The concurrent validity was satisfactory for three of the four constructs, indicated by the positive association between the constructs “self-efficacy” and “perceived social support” and loneliness, and the negative association between the construct “subjective norm” and loneliness.

For the development of the LLS the outcome model for health promotion of (Nutbeam 1998) was used as conceptual framework. In this model health literacy refers to the personal, cognitive and social skills that enable individuals to gain access to, understand, and use information. This information is assumed to change behavioural determinants such as knowledge, attitudes, motivations and self-efficacy related to a defined health promoting behaviour. According to Nutbeam (2000), these behavioural determinants can be regarded as measurable outcomes of health education. Further, Nutbeam (2009) ascertained a growing awareness of content and context specific literacy. The developed LLS integrated these two visions by including the constructs self-efficacy, subjective norm and perceived social support tailored to the topic loneliness in the local context of the *Healthy Ageing* programme.

A strength of the development procedure of the LLS is the structured approach of identifying causes of loneliness and related behavioural determinants. We combined theoretical evidence about causes of loneliness and general behavioural (change) theories with practical experiences of local policy makers, health and welfare workers and representatives of the target group. Furthermore, PCA and internal consistency analysis were used to affirm the scale structure, reduce the number of items, and assess the internal consistency reliability of the constructs. PCA with oblique rotation delivered best interpretable component solution and was therefore presented in this paper. The four component solution appeared to be quite robust as the components, respectively factors, were also found when the analysis is repeated with Common Factor Analysis (Floyd and Widaman 1995; Suhr 2005) and with orthogonal (varimax) instead of oblique (oblimin) rotation procedures. Only two items did not have one dominant component respectively factor. Finally, we evaluated the concurrent validity of the LLS by studying cross-sectionally the associations between the loneliness literacy constructs and loneliness in a heterogeneous study population. The regression analysis showed that this association was significant for three of the four constructs, namely “self-efficacy”, “perceived social support”, and “subjective norm”. As the LLS is a newly developed short-term indicator, it would be important for further research to investigate the predictive validity of the LLS on top of concurrent validity by use of a prospective study. In addition, it is recommended to confirm the hypothesized association between the loneliness literacy constructs and the target behaviours in a next study. Furthermore, in the current study we selected a heterogeneous study population in order to maximise variation. In a next study one might select a more representative sample to allow extrapolation of loneliness literacy estimates to the general elderly population.

PCA allocated the scale items in the theoretically defined constructs “knowledge” and “self-efficacy”, however the theoretical construct “motivation” was split into “perceived social support”, “subjective norm” and “motivation”.

The importance of including the construct “self-efficacy” in the model was affirmed by PCA and regression analyses. “Self-efficacy” was, compared with the other constructs, most strongly associated with loneliness in the univariate as well as the multivariate regression model. This association remained stable after adjustment for the other constructs and confounders. Higher, meaning less favourable, self-efficacy scores were related to more severe loneliness, thus confirming our expectations.

The construct “perceived social support” encompassed the social support experienced by older individuals from their social environment in the past. Perceived social support might either encourage or discourage a person to participate in social activities or to search for professional help in the future. In line with our expectations, higher (less favourable) scores on the construct “perceived social support” were significantly associated with more loneliness, still after adjustment for the other constructs and confounders.

The construct “subjective norm” included items about the opinion of important others, which might encourage or discourage a person to stay or become socially active. In the regression analysis we found a negative association with loneliness, meaning that less favourable literacy scores were associated with less severe loneliness. This association might probably be explained by reverse causality. Persons in the social environment of a more severely lonely person probably express more often their concerns and try to convince this person to go out and meet other people.

The new construct “motivation” included items about the awareness of offered services, expected outcomes of using these services and intention to use the service in case one would feel lonely. All six items were related to the target behaviour “searching for support”. The construct “motivation” was not significantly associated with loneliness in the univariate as well as the multivariate analyses. The reason that motivation did not appear as individual predictor of loneliness in this study might origin in the formulation of the items which was probably too hypothetical. For example, for healthy, socially active and not lonely people it might be very hard to imagine how one would act if their situation would deteriorate after certain life-events. Therefore, it might be difficult to answer a question such as: “*If* I felt lonely, *I would* search for professional help to reduce these feelings.”

Finally, the construct “knowledge” was not included in the resulting model. As knowledge is seen as prerequisite to change other behavioural determinants it is a shortcoming that we cannot include the construct knowledge in our scale (Bartholomew et al. 2011c). Communication of factual knowledge aiming to improve knowledge of health risks and health services is indicated by Nutbeam (2000) as functional health literacy. With regard to the *Healthy Ageing* programme the focus was on the latter of these two, namely knowledge or awareness about the existence of health services and opportunities for social engagement. Unfortunately, it appeared that the knowledge items of the LLS were difficult to answer, as indicated by the high number of missing values on these items and respondents’ notes. This implies that persons who are not lonely and are socially active do not (yet) experience a need for the services and activities listed in the questions and thus are not aware of their existence, which resulted in skipping questions. However, within *Healthy Ageing* several intervention activities aimed to increase awareness about the importance of maintaining a good social network. Instead of measuring factual knowledge about health services, we suggest to include scale items that focus on awareness about personal health benefits.

Finally, the suitability of the LLS to evaluate the *Healthy Ageing* programme, and thus to observe changes, depends on three aspects, namely: the scale sensitivity, the correctness of the hypothesised intervention logic model, and the content and magnitude of the implemented the intervention activities. With regard to the intervention activities, first the attention of the target group should be drawn and the delivered messages should be meaningful and acceptable to them before loneliness literacy can change. Besides, availability and accessibility of services and support resources are a prerequisite to ensure that improved loneliness literacy scores will result in more social engagement and searching for support. This is visualised by the box “environmental resources” in Fig. 1.

To summarize, to our knowledge this study is the first developing a short-term indicator for loneliness prevention. The concurrent validity of the LLS was satisfactory for three of the four constructs, indicated by the positive association between the constructs “self-efficacy” and “perceived social support” and loneliness, and the negative association between the construct “subjective norm” and loneliness. With the LLS we meet Nutbeams’ (2009) call-up for the development of health literacy indices that are tailored to specific health contents and contexts.

## Electronic supplementary material

Below is the link to the electronic supplementary material.

Supplementary material 1 (DOCX 23 kb)
