# Supplementary material for: Loneliness Literacy Scale: Development and Evaluation of an Early Indicator for Loneliness Prevention
Source: Soc Indic Res. 2013 Apr 3;116(3):989–1001. doi: 10.1007/s11205-013-0322-y (PMC3971461; doi:10.1007/s11205-013-0322-y)
Supplement: Supplementary file 1 — Supplementary material 1 (DOCX 23 kb) [file 11205_2013_322_MOESM1_ESM.docx]

**Supplementary material 1 - Loneliness Literacy Scale: Development and Evaluation of an Early Indicator for Loneliness Prevention**

**Items Loneliness Literacy Scale**

|  | **Item** | **Scale** | **Theoretical construct ^a^ /**  **target behaviour (1/2) ^b^** | |
| --- | --- | --- | --- | --- |
| motivation | If I have problems, a conversation with the elderly advisor helps me to solve my problems | (fully) agree – (fully) disagree | Attitudinal belief (expected outcome) | 2 |
|  | Meetings for bereavement are offered in my municipality | (fully) agree – (fully) disagree | Awareness | 2 |
|  | In my municipality there are professionals who can help people who feel gloomy or lonely | (fully) agree – (fully) disagree | Awareness | 2 |
|  | A support group would help me to give ageing problems a place | definitely – definitely not | Attitudinal belief (expected outcome) | 2 |
|  | If I felt lonely, I would search for professional help to reduce these feelings | definitely – definitely not | Intention | 2 |
|  | If I lost my partner, I would follow a bereavement course | definitely – definitely not | Intention | 2 |
| self-efficacy | I feel self-efficacious enough to go to an activity on my own | (fully) agree – (fully) disagree | Self-efficacy belief | 1 |
|  | I am able do almost anything if I really want to | (fully) agree – (fully) disagree | Self-efficacy belief | 1/ 2 |
|  | If I need help from others, I am able to arrange it myself | (fully) agree – (fully) disagree | Self-efficacy belief | 2 |
|  | In a group of friends/acquaintances, I speak up regularly | (fully) agree – (fully) disagree | Self-efficacy belief | 1/ 2 |
|  | I can manage in daily living as regards arranging transportation to activities | (fully) agree – (fully) disagree | Self-efficacy belief | 1 |
|  | I can manage in daily living as regards finding information | (fully) agree – (fully) disagree | Self-efficacy belief | 1/ 2 |
| perceived social support | I perceive my family’s opinion as important | (fully) agree – (fully) disagree | Motivation to comply (subjective norm) | 1 |
|  | My family is there for me if I ask for help | (fully) agree – (fully) disagree | Attitudinal belief (outcome expectation) | 2 |
|  | I perceive my neighbours’ opinion as important | (fully) agree – (fully) disagree | Motivation to comply (subjective norm) | 1 |
|  | My neighbours are there for me if I ask for help | (fully) agree – (fully) disagree | Attitudinal belief (outcome expectation) | 2 |
|  | I perceive my friends’ opinion as important | (fully) agree – (fully) disagree | Motivation to comply (subjective norm) | 1 |
|  | My friends are there for me if I ask for help | (fully) agree – (fully) disagree | Attitudinal belief (outcome expectation) | 2 |
| subjective norm | My family thinks it is important for me to participate in activities | (fully) agree – (fully) disagree | Normative belief | 1 |
|  | My neighbours think it is important for me to participate in activities | (fully) agree – (fully) disagree | Normative belief | 1 |
|  | My friends think it is important for me to participate in activities | (fully) agree – (fully) disagree | Normative belief | 1 |
|  | By participating in activities I remain among men | (fully) agree – (fully) disagree | Attitudinal belief (outcome expectation) | 2 |

|  | **Item** | **Scale** | **Theoretical construct /**  **target behaviour (1/2) ^a^** | |
| --- | --- | --- | --- | --- |
| not included | Do you know where you have to be to join sporting activities? ^b^ | Yes – no | Knowledge | 1 |
|  | Do you know where you have to be to join recreation activities? ^b^ | Yes – no | Knowledge | 1 |
|  | Do you know where you have to be to join courses? ^b^ | Yes – no | Knowledge | 1 |
|  | Do you know where you have to be to apply for a walking frame? ^b^ | Yes – no | Knowledge | 2 |
|  | Do you know where you have to be for financial support? ^b^ | Yes – no | Knowledge | 2 |
|  | Do you know where you have to be for assistance with household? ^b^ | Yes – no | Knowledge | 2 |
|  | Do you know where you have to be for transport services? ^b^ | Yes – no | Knowledge | 2 |
|  | Do you know where you have to be for help with administration? ^b^ | Yes – no | Knowledge | 2 |
|  | Do you know where you have to be for help in the house with little chores? ^b^ | Yes – no | Knowledge | 2 |
|  | Do you know where you have to be for meal services? ^b^ | Yes – no | Knowledge | 2 |
|  | If I had physical restrictions, I would apply for a mobility scooter, walking frame, hearing aids, etc. ^c^ | definitely – definitely not | Intention | 2 |
|  | I know where I have to be if I want to participate in activities in the neighbourhood ^c^ | (fully) agree – (fully) disagree | Knowledge | 1 |
|  | I enjoy participating in activities in the neighbourhood ^d^ | (fully) agree – (fully) disagree | Attitudinal belief | 1 |
|  | If I go to activities in the neighbourhood, I can forget my little discomforts and worries ^d^ | (fully) agree – (fully) disagree | Attitudinal belief (outcome expectation) | 1 |
|  | If I want to participate in an activity, nothing will stop me ^d^ | (fully) agree – (fully) disagree | Self-efficacy belief | 1 |
|  | In comparison to other elderly people, I perceive I can manage well in daily living ^d^ | (fully) agree – (fully) disagree | Self-efficacy belief | 1/ 2 |
|  | I can solve most of the problems in daily life myself ^d^ | (fully) agree – (fully) disagree | Self-efficacy belief | 1/ 2 |
|  | Normally, I take the initiative and introduce myself to an unknown person ^d^ | (fully) agree – (fully) disagree | Self-efficacy belief | 1/ 2 |
|  | I like to meet new people ^d^ | (fully) agree – (fully) disagree | Attitudinal belief | 1 |
|  | I perceive it as important to stay among men ^d^ | (fully) agree – (fully) disagree | Attitudinal belief | 1 |
|  | I can manage in daily living as regards applying for certain facilities ^d^ | (fully) agree – (fully) disagree | Self-efficacy belief | 2 |

^a^ Target behaviours: 1) becoming or staying social engaged; 2) searching for support

^b^ Excluded because of 2-point Likert scale items with a lot of missing values

^c^ Excluded because item did not load on any of the components (factor loading <0.4)

^d^ Improvement Cronbach’s coefficient α after exclusion of item; or exclusion of item to reduce number of items within component with minor reduction of Cronbach’s coefficient α

Note: Items loosely translated from Dutch into English for this article. A more precise translation would be required for use in practice.
